# Supplementary material for: Structurally heterogeneous ribosomes cooperate in protein synthesis in bacterial cells
Source: Nat Commun. 2025 Mar 20;16:2751. doi: 10.1038/s41467-025-57955-8 (PMC11926189; doi:10.1038/s41467-025-57955-8)
Supplement: Supplementary file 1 — Supplementary Information [file 41467_2025_57955_MOESM1_ESM.pdf]

SUPPLEMENTARY INFORMATION

TITLE

Heterogeneous ribosomes cooperate in protein synthesis in bacterial cells

AUTHORS

Karla Helena-Bueno[1,*] Sophie Kopetschke[2,*], Sebastian Filbeck[2], Lewis I. Chan[1], Sonia Birsan[1], Arnaud Baslé[1], Maisie Hudson[1], Stefan Pfeffer[2,&], Chris H. Hill[3,4,5,&], Sergey V. Melnikov[1,&]

AFFILIATIONS

[1] Biosciences Institute, Newcastle University, Newcastle upon Tyne, UK
[2] Centre for Molecular Biology, Heidelberg University, Heidelberg, Germany
[3] York Structural Biology Laboratory, University of York, York, UK
[4] York Biomedical Research Institute, University of York, York, UK
[5] Department of Biology, University of York, York, UK

[*]Contributed equally to this work

CORRESPONDENCE

s.pfeffer@zmbh.uni-heidelberg.de, chris.hill@york.ac.uk, sergey.melnikov@ncl.ac.uk

Supplementary Figures

Supplementary Fig. 1 | Focused classification of ribosomes isolated from stationary cultures of *P. urativorans*

Supplementary Fig. 2 | FSC curves and local resolution and orientation for the cryo-EM datasets

Supplementary Fig. 3 | Cryo-EM map and atomic structure of various bS20 segments

Supplementary Fig. 4 | ModelAngelo modelling of bS20

Supplementary Fig. 5 | Growth curve of actively growing *P. urativorans* at physiological conditions

Supplementary Fig. 6 | Cryo-ET data processing and focused classifications

Supplementary Fig. 7 | FSC curves and local resolution for the cryo-ET datasets

Supplementary Fig. 8 | 3D classification focused on the trigger factor binding site

Supplementary Tables

Supplementary Table 1 | FoldSeek identifies the protein as the ribosomal protein bS20

Supplementary Table 2 | Cryo-EM data collection, refinement and validation statistics

Supplementary Table 3 | Cryo-ET data collection, refinement and validation statistics

Supplementary Data

Supplementary Data 1 | Custom scripts used for cryo-ET data analysis

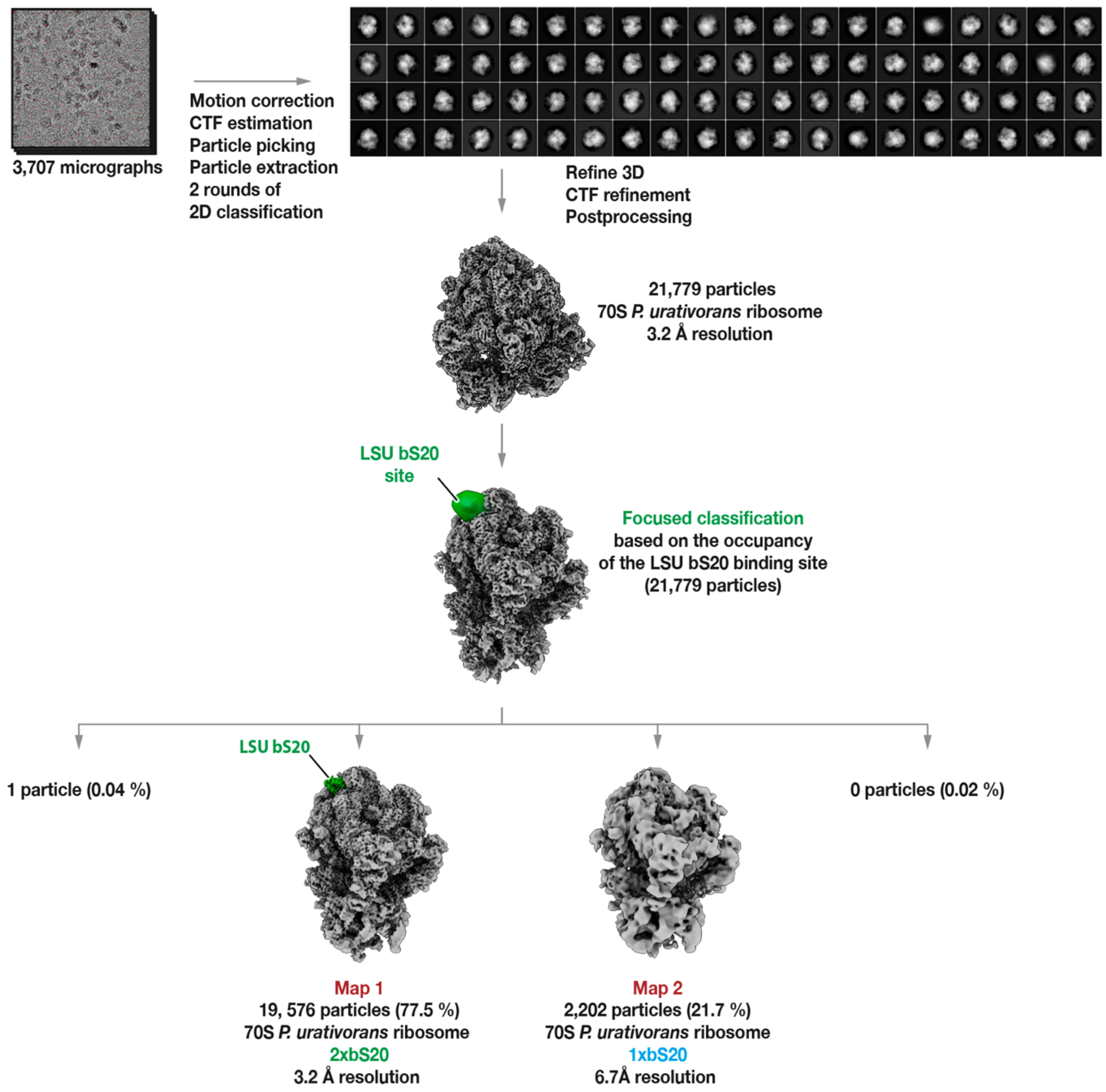

Supplementary Fig. 1 | Focused classification of ribosomes isolated from stationary cultures of *P. urativorans*. The pipeline shows a representative micrograph at 150,000 x magnification, 2D classes, 3D reconstructions and major steps of data processing using RELION 3.1 and RELION 5.0.

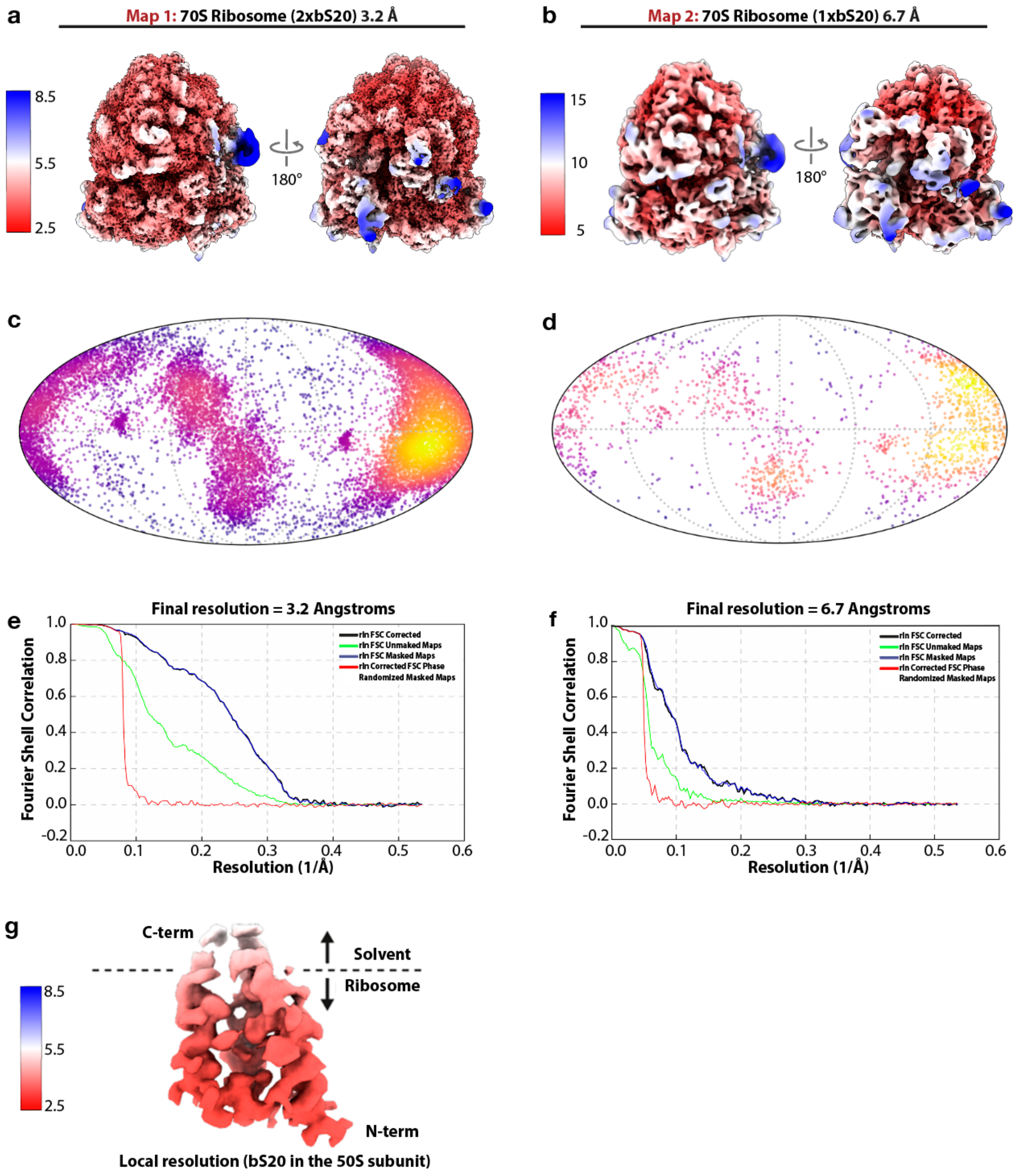

Supplementary Fig. 2 | Validation of cryo-EM maps of the *P. urativorans* ribosome bound with bS20. Panel descriptions refer to the *P. urativorans* 70S ribosome/bS20 maps: (**a**,**b**) Final cryo-EM maps, surface coloured by estimated local resolution as indicated in the heatmap key. Two orthogonal views are shown for each map corresponding to 2xbS20 ribosomes (**a**) and 1xbS20 ribosomes (**a**). (**c**,**d**) Angular distribution plots of particles in the final reconstructions, shown as a Mollweide projection for (**c**) 2xbS20 ribosomes and (**d**) 1xbS20 ribosomes. (**e**,**f**) Gold-standard Fourier shell correlation (FSC) curves for the final maps generated by RELION postprocessing for 2xbS20 ribosomes (**e**) and 1xbS20 ribosomes (**f**). Masked (blue), unmasked (green) and phase-randomised masked (red) plots are shown. (**g**) A segment of the cryo-EM map for 2xbS20 ribosomes corresponds to the bS20 molecule in the large ribosomal subunit and is coloured by local resolution.

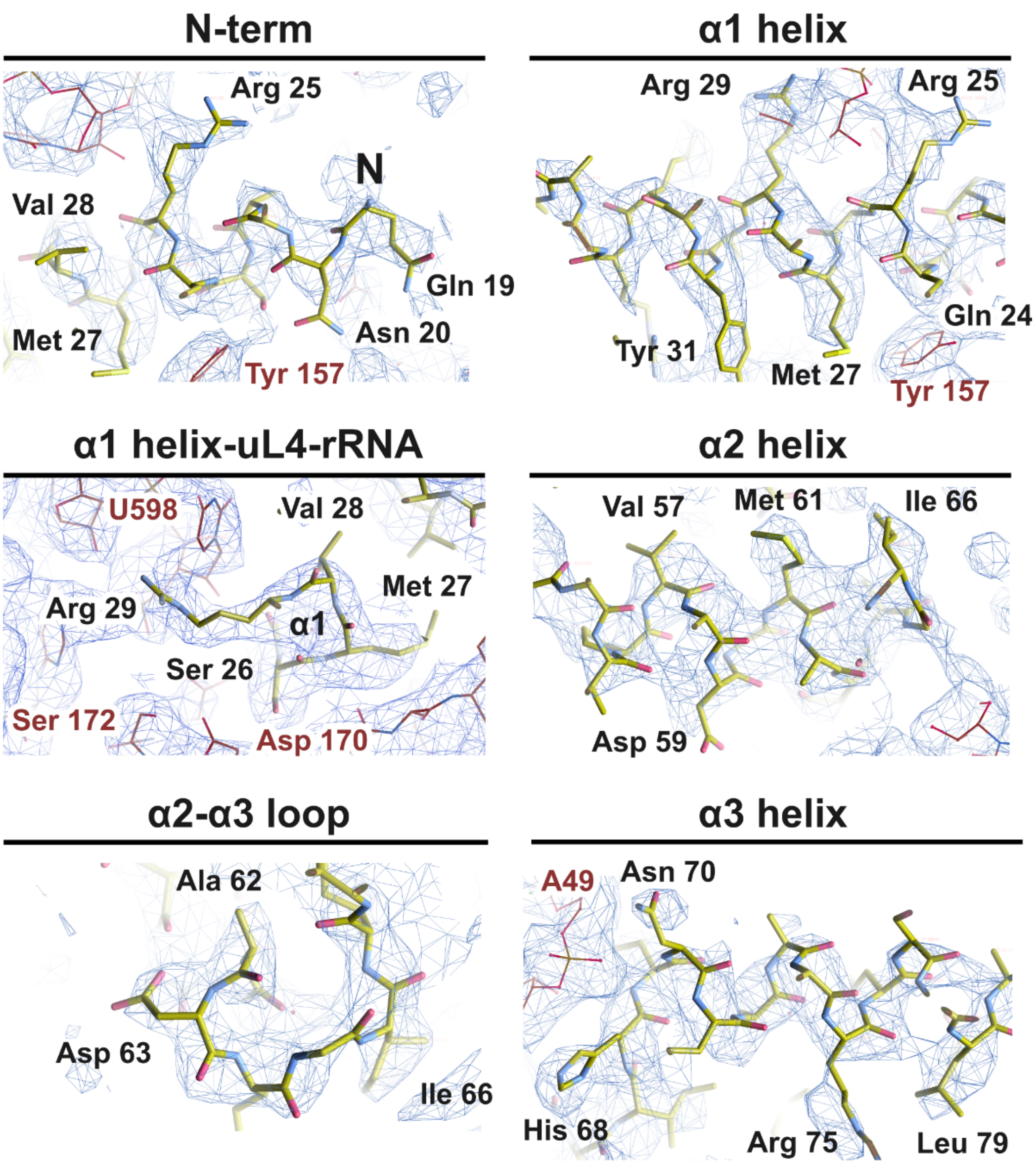

Supplementary Fig. 3 | Cryo-EM map and atomic structure of various bS20 segments. Close-up views illustrating the cryo-EM density (blue mesh) and atomic models (yellow sticks) for bS20 α1, α2 and α3 helices on the 50S subunit of *P. urativorans* ribosomes. Density is contoured at 2.7 – 2.3 σ. Red labels indicate residues of uL4 (e.g. Tyr157) or 23S rRNA (e.g. U598).

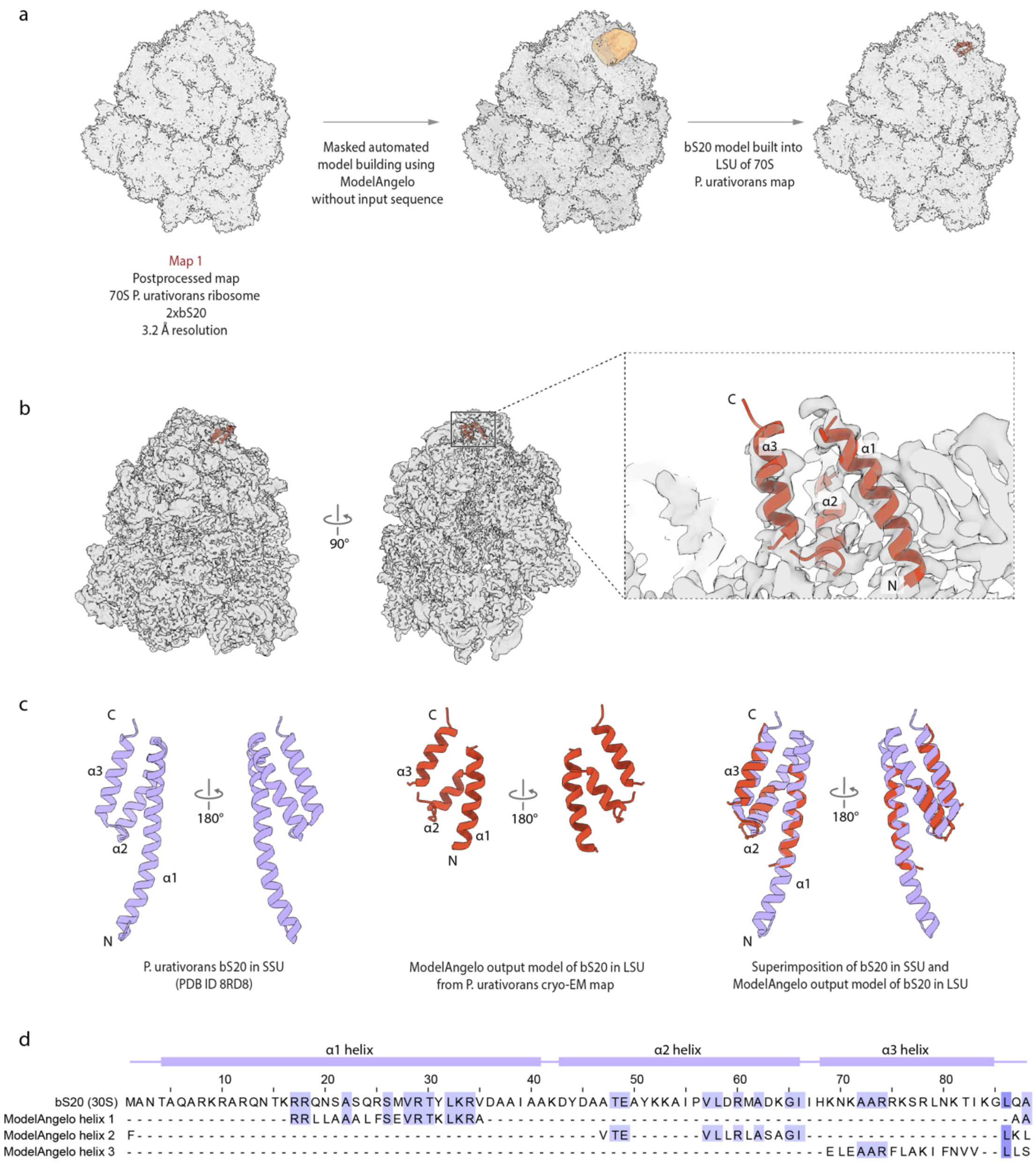

Supplementary Fig. 4 | ModelAngelo results confirm the identity of previously unassigned density in the large ribosomal subunit of *P. urativorans* ribosomes as ribosomal protein bS20. (a) Workflow of automated model building using ModelAngelo to identify the masked density shown in the large ribosomal subunit of *P. urativorans* ribosome. **(b)** ModelAngelo output model docked in cryo-EM maps filtered by local resolution of 70S *P. urativorans* ribosomes shown in two orthogonal views. Close-up view shows the overall fit of segments of bS20 built using ModelAngelo into the large subunit of *P. urativorans* ribosomes. (**c**) Comparison of the previously determined bS20 structure in the small subunit (SSU) to ModelAngelo output model in the large subunit (LSU) shows the structural similarity between these models consistent with RMSD values calculated for individual helices (Cα-atom RMSD α1 = 0.901 Å, RMSD α2 = 0.636 Å, RMSD α3 = 0.403 Å). **(d)** Sequence alignment shows conserved residues of bS20 from *P. urativorans* and output sequence from ModelAngelo modelled helices.

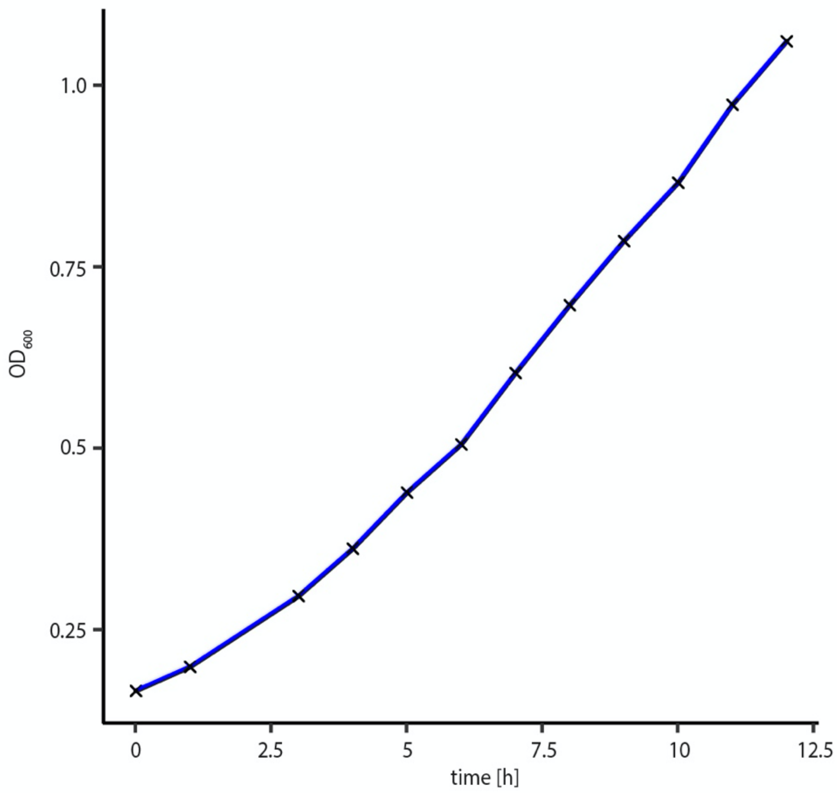

Supplementary Fig. 5 | Growth curve of actively growing *P. urativorans* at physiological conditions. *P. urativorans* was cultured at 19 °C and constant shaking. For OD_{600} measurements, 1 ml samples were taken every hour for 12 h in total. Samples for freezing were taken at an OD_{600} of 0.58. Comparable curves were reproduced 3 times. Source data are provided in a source data file.

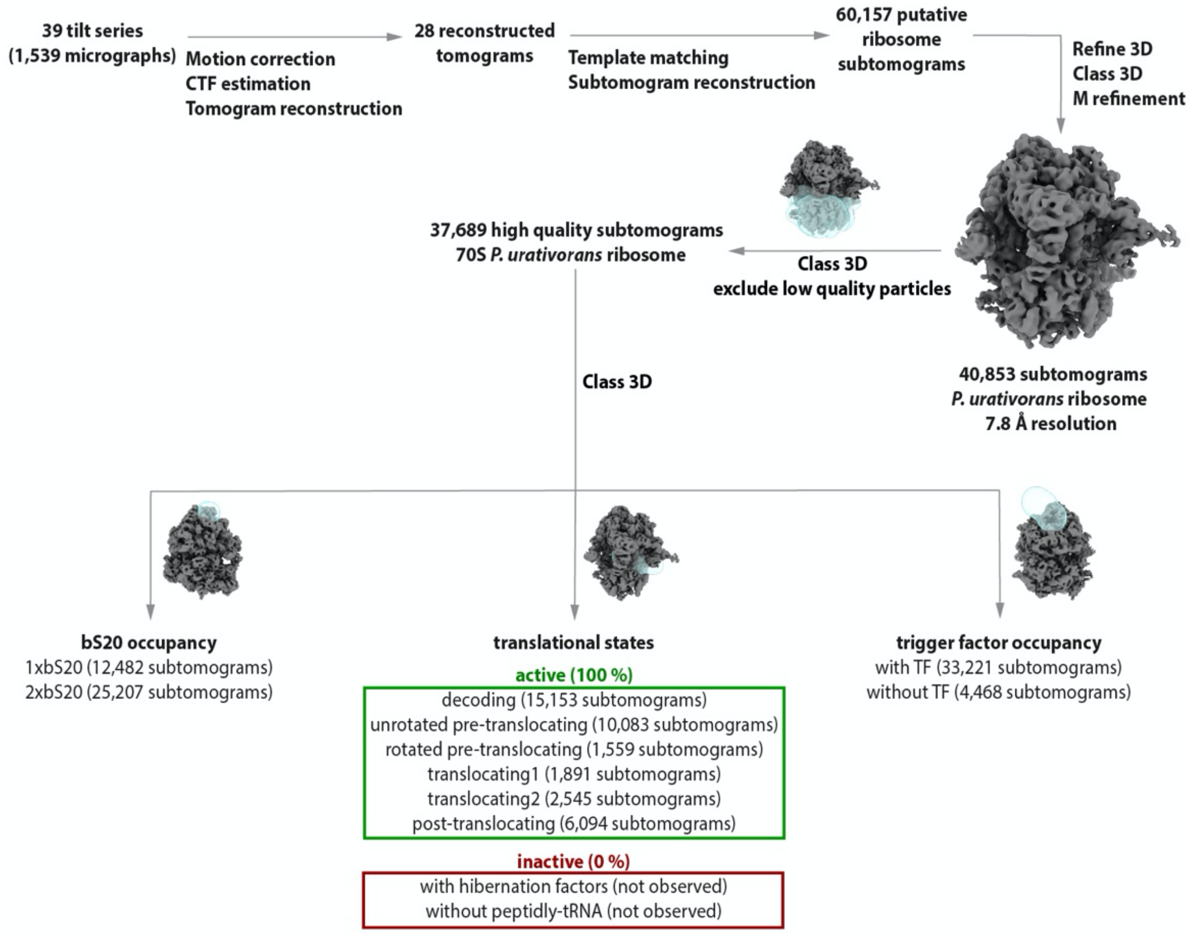

Supplementary Fig. 6 | Cryo-ET data processing scheme for *P. urativorans.* Tilt frame stacks were pre-processed using MotionCor2 and Warp and binned tomograms were reconstructed in Warp. Ribosomal particles were localized using template matching implemented in PyTom. Reconstructed subtomograms were aligned in RELION 3.1 and subjected to 3D classification. The resulting cryo-EM reconstruction was then used as input for local tilt-series refinement in M. Subsequently, optimized subtomograms were reconstructed and classified for quality and rotational state using a mask covering the 30S subunit. To identify translational states, rotated and unrotated ribosome populations were separately classified using a focus mask covering the factor binding site and the tRNA in A and P sites. To assess bS20 and Trigger Factor association, subtomograms were classified with focus masks for the bS20 and Trigger Factor binding sites on the 50S subunit.

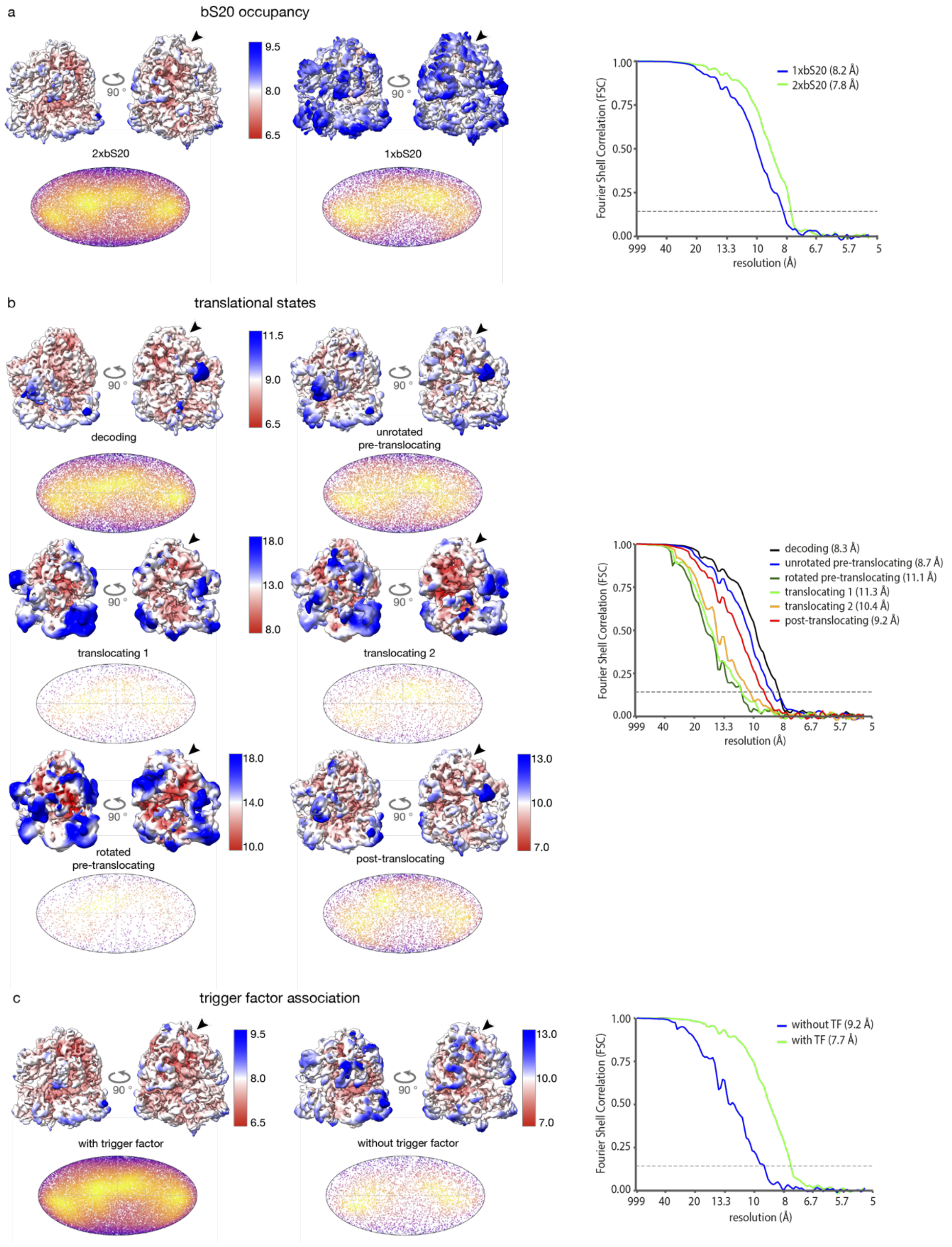

Supplementary Fig. 7 | FSC curves and local resolution for the cryo-ET datasets. Cryo-EM densities, surface coloured by estimated local resolution, angular distribution plots of the final reconstructions as Mollweide projections and FSC curves are shown for **(a)** 1xbS20 and 2xbS20 ribosomes, **(b)** different translational states and **(c)** ribosomes with and without trigger factor**.** Gold-standard mask corrected Fourier shell correlation (FSC) curves for the final densities were generated by RELION postprocessing. Arrow heads indicate the bS20 binding site on the 50S subunit. Source data are provided in a source data file.

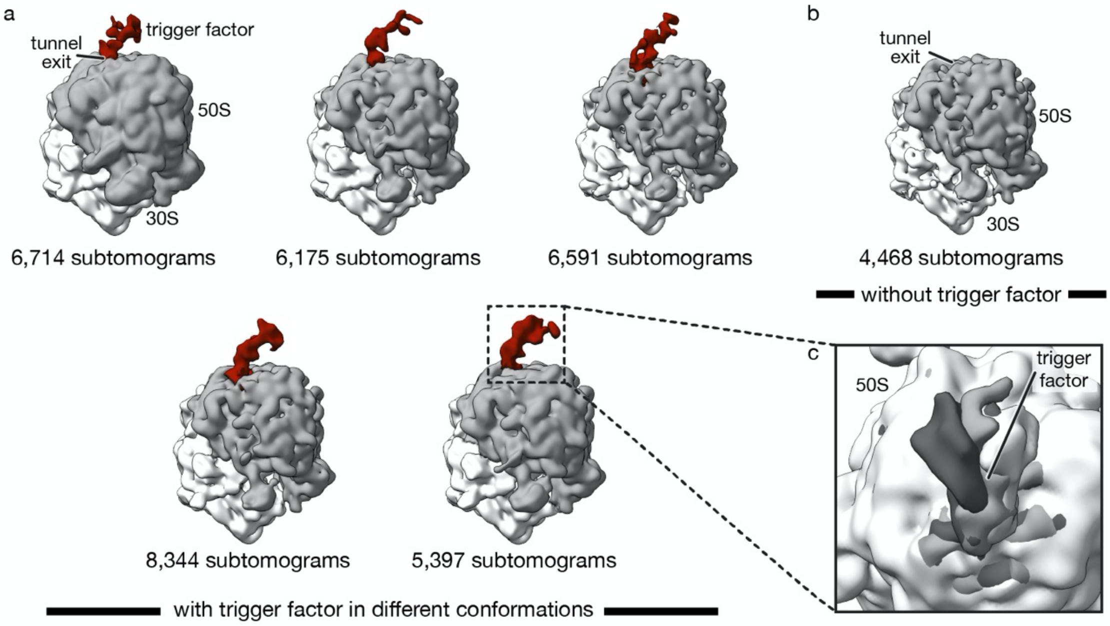

Supplementary Fig. 8 | 3D classification focused on the Trigger Factor binding site. Cryo-EM densities of all six 3D classes, **(a)** with and **(b)** without Trigger Factor bound to the ribosomal tunnel exit. **(c)** Superposition of three representative Trigger Factor-containing classes showing different conformations of trigger factor bound to the ribosome. For visualisation, all densities were low-pass filtered to 15 Å.

Supplementary Table 1 | FoldSeek identifies protein bS20 in the large ribosomal subunit of the 70S *P. urativorans* ribosomes. The table shows the top hits of FoldSeek search for proteins with similar three-dimensional fold to the protein identified in the cryo-EM map of *P. urativorans* ribosomes. The table indicates that in the predicted proteomes of *Psychrobacter* species, protein bS20 is the only protein with a significantly similar (and nearly identical) structure (as indicated by the E-value) to the C-alpha model of the protein identified in the cryo-EM map of *P. urativorans* ribosomes.

Target	Description	Scientific Name	Prob.	Seq. Id.	E-Value	Position in query
AF-A0A1B6NRS8-F1-model…	30S ribosomal protein S20	marine sediment metagenome	1.00	92.7	1.41e-6	1–69
AF-A0A2S7UYZ4-F1-model…	30S ribosomal protein S20	Psychrosphaera saromensis	1.00	61.1	1.16e-4	2–68
AF-A0A7U4CT18-F1-model…	30S ribosomal protein S20	Vibrio anguillarum M3	1.00	60.2	1.03e-4	1–68
AF-A0A285J3C1-F1-model_v4	30S ribosomal protein S20	Arsukibacterium tuosuense	1.00	58.8	9.64e-5	1–68
AF-A3M550-F1-model_v4	30S ribosomal protein S20	Acinetobacter baumannii AT…	1.00	73.1	9.06e-5	2–68
AF-A0A0F2R8Q7-F1-model…	30S ribosomal protein S20	Pseudomonas sp. BRH_c35	1.00	70.5	1.24e-4	1–68
AF-B4F2T3-F1-model_v4	30S ribosomal protein S20	Proteus mirabilis HI4320	1.00	55.8	1.91e-4	1–68
AF-A0A0I9SHW3-F1-model…	30S ribosomal protein S20	Pseudomonas sp. BICA1-14	1.00	70.5	1.49e-4	1–68
AF-A0A1Y5I1N2-F1-model_v4	30S ribosomal protein S20	Oleispira antarctica	1.00	56.7	1.59e-4	1–67
AF-A8G9L2-F1-model_v4	30S ribosomal protein S20	Serratia proteamaculans 568	1.00	57.3	2.30e-4	1–68
AF-A0A5N0TF93-F1-model_…	30S ribosomal protein S20	Wenzhouxiangella sp. W260	1.00	58.2	2.16e-4	2–68
AF-A7C0H2-F1-model_v4	30S ribosomal protein S20	Beggiatoa sp. PS	1.00	58.8	2.30e-4	1–68
AF-A0A0A2R704-F1-model_…	30S ribosomal protein S20	Morganella morganii	1.00	58.8	2.95e-4	1–68
AF-A0A5M9R030-F1-model…	30S ribosomal protein S20	Morganella psychrotolerans	1.00	55.8	2.95e-4	1–68
AF-A0A1H2Q0L5-F1-model…	30S ribosomal protein S20	Thiocapsa roseopersicina	1.00	59.4	3.78e-4	1–69
AF-A0A318W9C6-F1-model…	30S ribosomal protein S20	Thioalkalivibrio sp. ALE21	1.00	50.7	4.02e-4	2–68

Supplementary Table 2 | Cryo-EM data collection, refinement and validation statistics.

	P. urativorans **70S/2xbS20 (RELION, EMD-52036) (PDB 9HC4)**	***P. urativorans*** **70S/1xbS20 (RELION, EMD-52036)**
**Data collection and processing**		
Magnification	150k	150k
Voltage (kV)	200 kV	200kV
Electron exposure (e–/Å^2)	50	50
Defocus range (μm)	-0.5, -0.75, -1.0, -1.25	-0.5, -0.75, -1.0, -1.25
Pixel size (Å)	0.934	0.934
Symmetry imposed	C1	C1
Final particle images (no.)	19,576	2,202
Map resolution (Å)	3.2	6.7
FSC threshold	0.143	0.143
Map resolution range (Å)	2.89 – 7.67	4.28 – 14.23
**Refinement**		
Model resolution (Å)	3.1(masked)<br>3.1 (unmasked)	
FSC threshold	0.5	
Model resolution range (Å)	3.1-50	
Map sharpening *B* factor (Å^2)	-55.7	
Model composition		
Non-hydrogen atoms	136,434	
Protein residues	5,606	
Nucleotides	4,323	
B factors (Å^2) (min/max/mean)		
Protein	3.65/97.81/31.34	
Nucleotide	0.78/97.92/30.50	
R.m.s. deviations		
Bond lengths (Å)	0.002	
Bond angles (°)	0.531	
Validation		
MolProbity score	1.34	
Clashscore	5.90	
Poor rotamers (%)	0.00	
Ramachandran plot		
Favoured (%)	97.93	
Allowed (%)	2.03	
Disallowed (%)	0.04	

Supplementary Table 3 | Cryo-ET data collection and processing.

Data collection and processing	70S ribosome Average (EMD-52351)	70S 1xbS20 (EMD-52352)	70S 2xbS20 (EMD-52354)	70S With TF	70S without TF
Magnification	33 k	33 k	33 k	33 k	33 k
Voltage (kV)	300 kV	300 kV	300 kV	300 kV	300 kV
Electron exposure (e–/Å^2)	136	136	136	136	136
Defocus range (µm)	-3.0 to -6.0	-3.0 to -6.0	-3.0 to -6.0	-3.0 to -6.0	-3.0 to -6.0
Pixel size (Å)	2.589	2.589	2.589	2.589	2.589
Symmetry imposed	C1	C1	C1	C1	C1
Final particle images (no.)	37,689	12,482	25,207	33,221	4,468
Map resolution (Å)	7.8	8.2	7.8	7.7	9.2
FSC threshold	0.143	0.143	0.143	0.143	0.143
Map resolution range (Å)	5.6 – 12.3	7.0 – 12.8	6.6 – 9.6	6.6 – 10.5	7.4 – 14.0

Data collection and processing	Decoding	Unrotated pre-translocating	Rotated pre-translocating
Magnification	33 k	33 k	33 k
Voltage (kV)	300 kV	300 kV	300 kV
Electron exposure (e–/Å^2)	136	136	136
Defocus range (µm)	-3.0 to -6.0	-3.0 to -6.0	-3.0 to -6.0
Pixel size (Å)	2.589	2.589	2.589
Symmetry imposed	C1	C1	C1
Final particle images (no.)	15,153	10,083	1,559
Map resolution (Å)	8.3	8.7	11.1
FSC threshold	0.143	0.143	0.143
Map resolution range (Å)	6.7 – 14.0	7.2 – 12.6	8.4 – 22.0

Data collection and processing	Translocating 1	Translocating 2	Post-translocating
Magnification	33 k	33 k	33 k
Voltage (kV)	300 kV	300 kV	300 kV
Electron exposure (e–/Å^2)	136	136	136
Defocus range (µm)	-3.0 to -6.0	-3.0 to -6.0	-3.0 to -6.0
Pixel size (Å)	2.589	2.589	2.589
Symmetry imposed	C1	C1	C1
Final particle images (no.)	1,891	2,545	6,094
Map resolution (Å)	11.3	10.4	9.2
FSC threshold	0.143	0.143	0.143
Map resolution range (Å)	8.4 – 24.0	8.4 – 21.0	7.8 - 16
