# Supplementary material for: Structurally heterogeneous ribosomes cooperate in protein synthesis in bacterial cells
Source: Nat Commun. 2025 Mar 20;16:2751. doi: 10.1038/s41467-025-57955-8 (PMC11926189; doi:10.1038/s41467-025-57955-8)
Supplement: Supplementary file 2 — Description of Additional Supplementary Files [file 41467_2025_57955_MOESM2_ESM.pdf]

Description of Additional Supplementary Files

File name: Supplementary Data 1
Description: Custom scripts used for cryo-ET data analysis.
