# Supplementary material for: Structurally heterogeneous ribosomes cooperate in protein synthesis in bacterial cells
Source: Nat Commun. 2025 Mar 20;16:2751. doi: 10.1038/s41467-025-57955-8 (PMC11926189; doi:10.1038/s41467-025-57955-8)
Supplement: Supplementary file 4 — Reporting Summary [file 41467_2025_57955_MOESM4_ESM.pdf]

nature portfolio

Corresponding author(s): Sergey V. Melnikov
Chris H. Hill
Stefan Pfeffer

Last updated by author(s): Feb 18, 2025

Reporting Summary

Nature Portfolio wishes to improve the reproducibility of the work that we publish. This form provides structure for consistency and transparency in reporting. For further information on Nature Portfolio policies, see our Editorial Policies and the Editorial Policy Checklist.

Statistics

For all statistical analyses, confirm that the following items are present in the figure legend, table legend, main text, or Methods section.

n/a	Confirmed	
☐	☒	The exact sample size (*n*) for each experimental group/condition, given as a discrete number and unit of measurement
☐	☒	A statement on whether measurements were taken from distinct samples or whether the same sample was measured repeatedly
☒	☐	The statistical test(s) used AND whether they are one- or two-sided<br>*Only common tests should be described solely by name; describe more complex techniques in the Methods section.*
☒	☐	A description of all covariates tested
☒	☐	A description of any assumptions or corrections, such as tests of normality and adjustment for multiple comparisons
☐	☒	A full description of the statistical parameters including central tendency (e.g. means) or other basic estimates (e.g. regression coefficient) AND variation (e.g. standard deviation) or associated estimates of uncertainty (e.g. confidence intervals)
☒	☐	For null hypothesis testing, the test statistic (e.g. *F*, *t*, *r*) with confidence intervals, effect sizes, degrees of freedom and *P* value noted<br>*Give P values as exact values whenever suitable.*
☒	☐	For Bayesian analysis, information on the choice of priors and Markov chain Monte Carlo settings
☒	☐	For hierarchical and complex designs, identification of the appropriate level for tests and full reporting of outcomes
☒	☐	Estimates of effect sizes (e.g. Cohen's *d*, Pearson's *r*), indicating how they were calculated

Our web collection on statistics for biologists contains articles on many of the points above.

Software and code

Policy information about availability of computer code

Data collection

The cryo-EM data was collected using the Smart EPU software for single particle analysis by Thermofisher Scientific.
cryo-ET data was collected using the Tomo5 (version 5.5.0.1941) software for tilt series acquisition by Thermofisher Scientific on a Krios TEM with a Gatan K3 camera operated by Gatan Microscopy Suite (version 3.32) .
The bacterial growth curves were recorded using an Ultrospec 2100 pro by Amersham Biosciences.

Data analysis

The models were created using Coot v0.8.9.2
The models were analyzed and visualized using ChimeraX v1.8 and UCSF Chimera 1.16
The cryo-EM data was processed using RELION v3.1 and v5.0
The models were refined using Phenix v1.20.1-4487
Tilt Series Alignment IMOD 4.11.19
Motion Correction in MotionCor 1.0.5
Tilt Series Reconstruction and CTF estimation Warp 1.0.9
template matching in PyTom 0.971
Local Tilt Series alignment M 1.0.9
Polysome tracing in python 3 (available with the article)
Plots were generated in R 4.3.2

For manuscripts utilizing custom algorithms or software that are central to the research but not yet described in published literature, software must be made available to editors and reviewers. We strongly encourage code deposition in a community repository (e.g. GitHub). See the Nature Portfolio guidelines for submitting code & software for further information.

Data

Policy information about availability of data

All manuscripts must include a data availability statement. This statement should provide the following information, where applicable:

- Accession codes, unique identifiers, or web links for publicly available datasets
- A description of any restrictions on data availability
- For clinical datasets or third party data, please ensure that the statement adheres to our policy

The atomic coordinate file for the structure of P. urativorans ribosomes with two copies of bS20 generated in this study has been deposited in the Protein Data Bank (PDB) with the accession code 9HC4 [https://www.rcsb.org/structure/unreleased/9HC4]. The associated cryo-EM maps generated in this study were deposited to the Electron Microscopy Data Bank (EMDB) with the accession codes EMD-52036 [https://www.ebi.ac.uk/emdb/EMD-52036] (cryo-EM reconstruction of 2xbS20 ribosomes), EMD-52351 [https://www.ebi.ac.uk/emdb/EMD-52351] (subtomograms average of all ribosomes), EMD-52352 [https://www.ebi.ac.uk/emdb/EMD-52352] (subtomograms average of 1xbS20 ribosomes) and EMD-52354 [https://www.ebi.ac.uk/emdb/EMD-52354] (subtomograms average of 2xbS20 ribosomes). A representative tomogram has been deposited to the EMDB with the accession code EMD-52842 [https://www.ebi.ac.uk/emdb/EMD-52842]. Source data are provided with this paper.

Research involving human participants, their data, or biological material

Policy information about studies with human participants or human data. See also policy information about sex, gender (identity/presentation), and sexual orientation and race, ethnicity and racism.

Reporting on sex and gender	We did not study any human populations in our study.
Reporting on race, ethnicity, or other socially relevant groupings	We did not study any human populations in our study.
Population characteristics	We did not study any human populations in our study.
Recruitment	We did not study any human populations in our study.
Ethics oversight	We did not study any human populations in our study.

Note that full information on the approval of the study protocol must also be provided in the manuscript.

Field-specific reporting

Please select the one below that is the best fit for your research. If you are not sure, read the appropriate sections before making your selection.

☒ Life sciences ☐ Behavioural & social sciences ☐ Ecological, evolutionary & environmental sciences

For a reference copy of the document with all sections, see nature.com/documents/nr-reporting-summary-flat.pdf

Life sciences study design

All studies must disclose on these points even when the disclosure is negative.

Sample size	For cryo-EM and cryo-ET data, no statistical method was chosen to determine the sample size. The number of micrographs or tilt-series was chosen to obtain a number of particles sufficient to reconstruct a 3D density of the 70S ribosome.<br>Each cryo-EM map presented in this study was calculated by using 3,3707 micrographs used to reconstruct the three-dimensional volume of the ribosome from a total of 21,779 ribosomal particles.<br>28 tilt series were used for tomogram reconstruction. Initial cryo-ET maps were calculated form 60,175 particles. After multiple rounds of 3D Classification a final dataset of 40,853 ribosomal particles was reached. The final reconstructions reached resolution that was estimated by the Gold Standard FSC method.
Data exclusions	During cryo-EM and cryo-ET data processing particles that were allocated into poorly resolved classes were excluded, s is standard image processing practice in cryo-EM.For cryo-electron tomography analysis, tomograms were excluded based on alignment quality and performance in template matching attempts. Subtomogram selection after template matching was performed through 3D classification and data quality of the resulting 3D maps has been used as exclusion criterion.
Replication	cryo-ET data was acquired in two sessions using two independent biological replicates
Randomization	Positions for image acquisition in cryo-EM were selected based on the presence of the particles.
Blinding	Cryo-EM and Cryo-ET were not blinded because it is performed computationally.

Reporting for specific materials, systems and methods

We require information from authors about some types of materials, experimental systems and methods used in many studies. Here, indicate whether each material, system or method listed is relevant to your study. If you are not sure if a list item applies to your research, read the appropriate section before selecting a response.

Materials & experimental systems

n/a	Involved in the study
☒	☐ Antibodies
☒	☐ Eukaryotic cell lines
☒	☐ Palaeontology and archaeology
☒	☐ Animals and other organisms
☒	☐ Clinical data
☒	☐ Dual use research of concern
☒	☐ Plants

Methods

n/a	Involved in the study
☒	☐ ChIP-seq
☒	☐ Flow cytometry
☒	☐ MRI-based neuroimaging

Plants

Seed stocks	We did not study plants in our study.
Novel plant genotypes	We did not study plants in our study.
Authentication	We did not study plants in our study.
